# Diamond Quantum Sensing Revealing the Relation between Free Radicals and Huntington’s Disease

**DOI:** 10.1021/acscentsci.3c00513

**Published:** 2023-06-21

**Authors:** S. Fan, L. Nie, Y. Zhang, E. Ustyantseva, W. Woudstra, H. H. Kampinga, R. Schirhagl

**Affiliations:** † University Medical Center Groningen, Groningen University, Antonius Deusinglaan 1 9713AV Groningen, The Netherlands

## Abstract

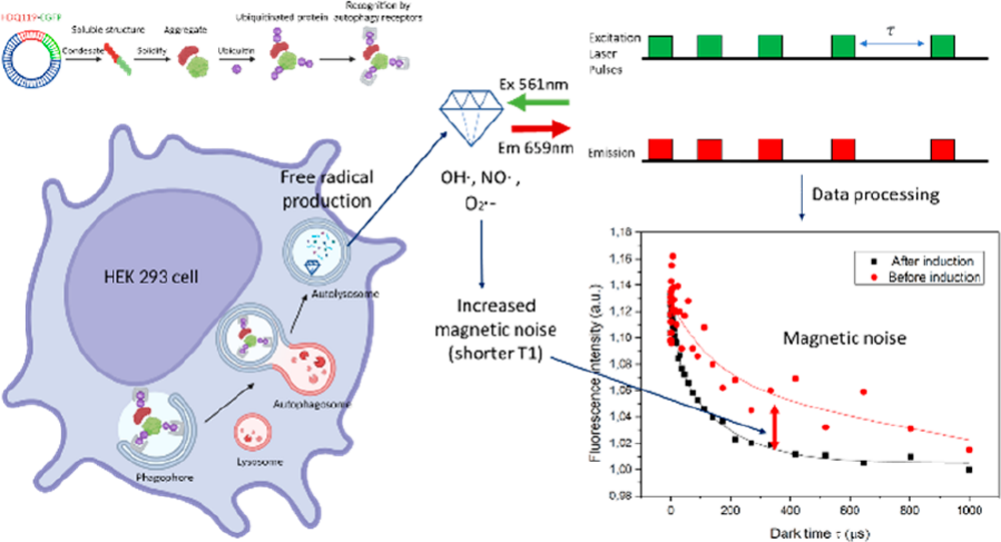

Huntington’s disease (HD) is a well-studied yet rare disease caused by a specific mutation that results in the expression of polyglutamine (PolyQ). The formation of aggregates of PolyQ leads to disease and increases the level of free radicals. However, it is unclear where free radicals are generated and how they impact cells. To address this, a new method called relaxometry was used to perform nanoscale MRI measurements with a subcellular resolution. The method uses a defect in fluorescent nanodiamond (FND) that changes its optical properties based on its magnetic surroundings, allowing for sensitive detection of free radicals. To investigate if radical generation occurs near PolyQ aggregates, stable tetracycline (tet)-inducible HDQ119-EGFP-expressing human embryonic kidney cells (HEK PQ) were used to induce the PolyQ formation and Huntington aggregation. The study found that NDs are highly colocalized with PolyQ aggregates at autolysosomes, and as the amount of PolyQ aggregation increased, so did the production of free radicals, indicating a relationship between PolyQ aggregation and autolysosome dysfunction.

## Abstract 2

Relaxometry and nanoscale MRI measurements reveals that free radicals are generated near PolyQ aggregates at autolysosomes. This is likely either a cause or consequence of the Huntington disease phenotype.

## Introduction

Huntington’s disease (HD) is a hereditary neurodegenerative disorder that causes a wide range of behavioral, cognitive, and physical symptoms.^1^ This autosomal dominant disease is caused by a CAG trinucleotide repeat expansion in exon 1 of the huntingtin gene (HTT).^2^ Repeat lengths above a critical threshold of 35 CAG triplets in HTT are defined as disease-causing alleles while in healthy individuals the length is only 18 repeats.^3^ The CAG repeat in HTT leads to an expansion of the polyglutamine (PolyQ) tract at the N-terminus of HTT, which can become an amyloid core and induce toxic protein aggregation.^4^ Autolysosomes are involved in the degradation of aggregated proteins (aggrephagy), but the failure of autolysosomal degradation can potentiate aggregate toxicity and related degeneration.^5^ As the PolyQ disease is caused by a pathogenic protein that possesses a clear amyloidogenic core, the expanded PolyQ tract, it is an excellent model for studying the pathological mechanism of protein aggregation-related diseases.^6^

To better understand the relation between redox imbalance and HD, our study used a fragment of exon 1 of the Huntington gene with 119 glutamines (HDQ 119), tagged with a green fluorescent protein (EGFP); the preparation of cells has been demonstrated before.^7^

While elevated oxidative stress and imbalanced redox signaling are known hallmarks of HD, their relation to neurodegeneration remains unclear.^8^ Pena-Sanchez et.al.^9^ found that the dysregulated glutathione metabolism could lead to redox imbalance in HD. Sbodio et.al.^10^ revealed that excessive oxidative stress perturbs signaling mediated by activating transcription factor 4 (ATF4), which is a master regulator of amino acid homeostasis. Prior studies have detected altered levels of antioxidant molecules and enzymes, but these methods are generally not specific to revealing the relationship between free radicals and HD. Further, these methods offer only limited spatial resolution due to diffusion of the dye molecules. Additionally, the dyes bleach over time, making it difficult to follow the change in reactive oxygen species (ROS). The reactions between the dye and ROS molecules are irreversible, measuring accumulated ROS production rather than the current status.

Herein, we use diamonds containing nitrogen-vacancy (NV) centers for quantum sensing to detect radicals. Such NV centers have already been used successfully for several nanoscale sensing applications in physics including the measurements of magnetic nanostructures, nanoparticles, or spin defects.^11−14^ NV centers in diamonds also allow measurements at extreme pressures or temperature.^15,16^ Also, their usefulness in biology has already been demonstrated. Davis et al.^17^ have, for instance, used NV centers to visualize spin labels in slices of fixed cells while Ermakova et al.^18^ have demonstrated the measurement of iron-containing proteins. Depending on the exact measurement mode, NV centers can also be used for nanoscale temperature measurements^15,19^ or to measure orientation.^20,21^ Recently, our group has shown that it is possible to use ensembles of NV centers in nanodiamonds to detect free radical generation on the nanoscale. Since then, the method has been applied in aging yeast cells,^22^ immune cells,^23,24^ and endothelial cells,^25^ during viral infection^26^ or during sperm cell maturation.^27^ Here we show the first detection of free radicals in autolysosomes during the induction of Huntington cells, offering new insights into the pathological mechanism of protein aggregation-related diseases.

## Materials and Methods

### Materials

The Fluorescent Nanodiamonds (FNDs) used in this study were purchased from Adamas Nanotechnologies in North Carolina, USA. These FNDs have a hydrodynamic diameter of 70 nm and contain over 300 NV^–^ centers. These particles were generated by high-temperature, high-pressure (HPHT) synthesis followed by irradiation with 3 MeV electrons at a fluence of 5 × 10^19^ e/cm^2^ and high-temperature annealing (above 700 °C). The manufacturer cleaned the particles in oxidizing acids, resulting in oxygen-terminated FNDs, which were characterized previously.^28,29^ We used FNDs of this size since relatively large particles have higher fluorescent countsand can thus be tracked easier, resulting in a good signal-to-noise ratio. In addition, each measurement is an average of all the NV^–^ centers which improves reproducibility.^23^ Even larger particles would not be ideal either since then NV centers would be too far away from the surface to sense free radicals in their surrounding. FNDs are also biocompatible and show stable fluorescence when taken up by cells.^30,31^

### Cell Culture

Stable tetracycline (tet)-inducible HDQ119-EGFP-expressing cells (HEK PQ) were produced as shown in references (32) and (33). The cells were cotransfected with pcDNA5/FRT/TO HDQ119-EGFP and the flippase (Flp) recombinase expressing plasmid pOG44 and selected with 100 mg/mL hygromycin.

Cultures were maintained at 37 °C and 5% CO_2_ in a humidified incubator in Dulbecco’s Modified Eagle Medium (DMEM, Invitrogen/Gibco) with 10% fetal bovine serum (FBS, Greiner bio-one) and optionally 100 units/mL penicillin and 100 μg/mL streptomycin (Invitrogen). The cells were subcultured twice a week at a dilution of about 1:10, trypsinized with Trypsin/EDTA (Invitrogen). For HEK 293 wild type cells (HEK WT), once per week, fresh Blasticidine (Invitrogen) and fresh Zeocin (Invitrogen) were added to the culture medium to final concentrations of 5 and 100 μg/mL, respectively. For culturing HEK cells containing PolyQ plasmids (HEK PQ), fresh Blasticidine (Invitrogen, 5 μg/mL) and Hygromycin B (Invitrogen, 100 μg/mL) were added to the culture medium once per week. To induce HEK PQ cells to stably express polyQ protein (HEK PQi), 1 μg/mL tetracycline (Invitrogen) was added to the medium 24, 36, or 48 h before the detection. As HEK 293 cells do not adhere very well to the glass-bottomed Petri dishes, 0.2% gelatin (Merck) was applied to coat the dishes prior to introducing HEK 293 cells.

### Cell Viability Test

We used the CellTiter-Glo Luminescent Cell Viability Assay (Promega) to determine the number of viable cells in culture. The assay is based on the quantification of ATP and is an indicator of metabolically active cells. To conduct the assay, HEK 293 cells were seeded (50,000 cells/well) in clear flat-bottom 96-well plates. We discarded the cell culture medium and rinsed the cells once with phosphate-buffered saline (PBS). Then, cells were incubated with 10 μg/mL FNDs or 5% dimethyl sulfoxide (DMSO) (as a positive control) for 24 h. After incubation, we equilibrated the plate and its contents to room temperature for approximately 30 min. We added 100 μL of CellTiter-Glo 2.0 Reagent to 100 μL of medium-containing cells. Then, we mixed for 2 min on an orbital shaker to induce cell lysis and allowed the plate to incubate at room temperature for 10 min to stabilize the luminescent signal. The luminescence was determined using a FLUOstar Omega Microplate Reader (BMG Labtech, De Meern, The Netherlands). Untreated cells were used as negative control.

### PolyQ expression in cells

Cells were seeded (100,000 cells/mL) in 35 mm glass-bottom cell culture dishes (Greiner bio-one). To induce them to express PolyQ proteins, they were incubated with 1 μg/mL of tetracycline for 24, 36, and 48 h, respectively. After incubation, the medium was discarded, and cells were rinsed once with PBS. Subsequently, the cells were fixed with 4% formaldehyde (PFA) for 10 min, stained with 4′,6-diamidino-2-phenylindole (DAPI, for staining the nucleus). Confocal images were acquired with a Zeiss 780 laser-scanning microscope (Zeiss, Jena, Germany). PolyQ protein was tagged with GFP which allowed us to visualize PolyQ expression and aggregation. DAPI and GFP were imaged at ex/em = 358/461 and ex/em = 495/510 nm, respectively. Confocal images were analyzed using the FIJI software (http://fiji.sc/) to measure the average GFP intensity of around 100 random cells. Integrated density (Int Den) was analyzed as the sum of the values of the pixels in the image or selection per cell to indicate the amount of GFP. A control group was used to subtract the background for the other groups.

### Protein Extraction for FTA and Western Blot

Frozen cell pellets were lysed in 230 μL of the lysis buffer (50 mM TRIS-HCl pH 7.4, 100 mM NaCl, 1 mM MgCl_2_, 0.5% SDS, EDTA-free complete protease inhibitors cocktail (Roche), and 50 units/mL Denarase (c-LEcta)). Then they were incubated on ice for 30 min with occasional vortexing. After incubation, the SDS concentration was increased to 2%. Protein concentrations were measured with a DC protein assay (Bio-Rad), equalized using dilution buffer (50 mM TRIS-HCl pH 7.4, 100 mM NaCl, 1 mM MgCl_2_, 2% SDS, 50 mM DTT), boiled for 5 min, and stored at −20 °C.

### Filter Trap Assay

For filter a trap assay (FTA), samples were diluted 5-fold in a dilution buffer. Then, both original (1×) and dilute (0.4×) samples were applied onto a 0.2 μm pore Cellulose acetate membrane, prewashed with FTA buffer (10 mM TRIS-HCl pH 8.0, 150 mM NaCl, 0.1% SDS). The membrane was washed under mild suction three times with FTA buffer, blocked in 10% nonfat milk, and blotted with anti-GFP/YFP antibody (mouse monoclonal IgG2, JL-8, Clontech, Cat no. 632381) at 1:5000 dilution overnight at 4 °C on a rocking platform. After, the membrane was incubated with an HPR-conjugated secondary antibody (GE Healthcare, Cat no. NXA931) and visualized with enhanced chemiluminescence using a ChemiDoc Imaging System (Bio-Rad). Signal intensities were measured by ImageJ, and the results of the two dilutions were averaged. Values were normalized to the 48 h sample, analyzed with GraphPad Prism, and plotted in a graph. The resulting graph represents the average of three separate experiments.

### Western Blot Analysis

For Western Blot (WB) analysis, sample aliquots were mixed with 4× sample buffer (50 mM TRIS-HCl pH 6.7, 2% SDS, 10% glycerol, 12.5 mM EDTA, and 0.02% Bromophenol blue). Samples were loaded on a 10% SDS-PAGE gel and ran at 90 V. Proteins were transferred to a nitrocellulose membrane (Schleicher and Schuell, PerkinElmer, Waltham, MA, USA), blocked in 10% nonfat milk, and blotted with primary anti-GAPDH antibody (1:5000, mouse monoclonal clone GAPDH-71.1, Sigma, Cat no. G8795), anti-GFP antibody (same as used for FTA) overnight at 4 °C on a rocking platform. After that, the membrane was incubated with an HPR-conjugated secondary antibody (GE Healthcare, Cat no. NXA931 and NA934) and visualized with enhanced chemiluminescence using a ChemiDoc Imaging System (Bio-Rad). Signal intensities were measured by ImageJ. Expression of soluble polyQ119-GFP, DNAJB6-V5 was normalized to the GAPDH, analyzed with GraphPad Prism. The resulting graph represents an average of three separate experiments.

### Cellular Uptake of FNDs

Cells were seeded (100,000 cells/mL) in 35 mm glass-bottom cell culture dishes (Greiner bio-one) and incubated with 10 μg/mL of FNDs for 5, 10, 15, 20, and 25 h, respectively. Then, cells were fixed with 4% PFA for 10 min and stained with DAPI and fluorescein phalloidin (FITC- phalloidin for staining F-actin). Confocal images were acquired with a Zeiss 780 laser-scanning microscope (Zeiss, Jena, Germany). FNDs were detected at ex/em = 561/659 nm; DAPI and FITC were imaged at 358/461 nm and 495/510 nm, respectively.^34^

Diffraction PSF 3D and iterative deconvolve 3D plugins of FIJI were used to improve the signal-to-noise ratio before counting.

For each cell type (HEK-WT, HEK PQ, HEK PQi), around 100 random cells were selected and analyzed. Z-stack confocal images containing the whole cell volume were acquired and deconvolved. The numbers of FNDs per cell were then analyzed using the 3D object counter plugin of FIJI. A size filter was set to 8 and the threshold was set to 40. This number was determined earlier from a control group to separate the FND signal from the background. This was the smallest number, where the signal from the control group was 0.

### Subcellular Location of FNDs

To track the location of diamond particles inside HEK 293 cells after 5 h of incubation, Lysoview 405 (Biotum) was used to label lysosomes. The cells were initially incubated with 10 μg/mL of FNDs for 5 h and then washed with PBS three times to avoid continuous uptake of FNDs. This washing step allowed tracking of the location of FNDs that had already been endocytosed by the cells. The location of FNDs within the 5-hour time frame was tracked by checking the starting time point (5h+0h) and the end time point (5h+5h). After that, lysoview 405 was added to the cells at a final concentration of 1 μg/mL, and the cells were incubated for 10 min. Live cells were then imaged by using a Zeiss 780 laser-scanning microscope. Around 60 random cells from each of three independent experiments were selected for each cell type. FNDs were detected at ex/em = 561/659 nm, lysoview 405 was imaged at ex/em = 358/461 nm, and GFP was imaged at 495/510 nm. To improve the signal-to-noise ratio, the obtained Z-stack images were deconvoluted by Diffraction PSF 3D and iterative deconvolve 3D plugins of FIJI. Then the JAcoP plugin^34^ in FIJI (https://imagej.nih.gov/ij/plugins/track/jacop.html) was used to analyze whether FNDs colocalized with lysoview 405 (lysosome) or GFP (PolyQ). The Manders’ Coefficient (MC) indicated the fraction of FNDs in autolysosomes or the fraction of FNDs in PolyQ-GFP aggregates.^35^

### Free Radical Measurements in HEK 293 Cells by T1 Measurement

A home-built magnetometry setup was used for T1 measurements as described previously.^36−38^ Cells were seeded (100,000 cells/mL) in 35 mm glass bottom Petri dishes and incubated overnight at 37 °C and 5% CO_2_.

To investigate free radical generation in lysosomes, HEK PQ cells were induced with 1 μg/mL tetracycline for 24, 36, or 48 h, respectively. Then, the inducer was washed away, FNDs were added to the cell culture medium, and we incubated for another 5 h. After incubating with FNDs, cells were washed with PBSm and PBS was replaced with a culture medium. T1 measurements were performed 3 times for every group. As controls, T1 measurements were also performed on tetracycline-induced HEK WT cells or FNDs outside cells. For the second control, tetracycline was dissolved in cell culture medium; then, it was added to FNDs to test if tetracycline interfered with T1 measurements.

**Figure 1:**
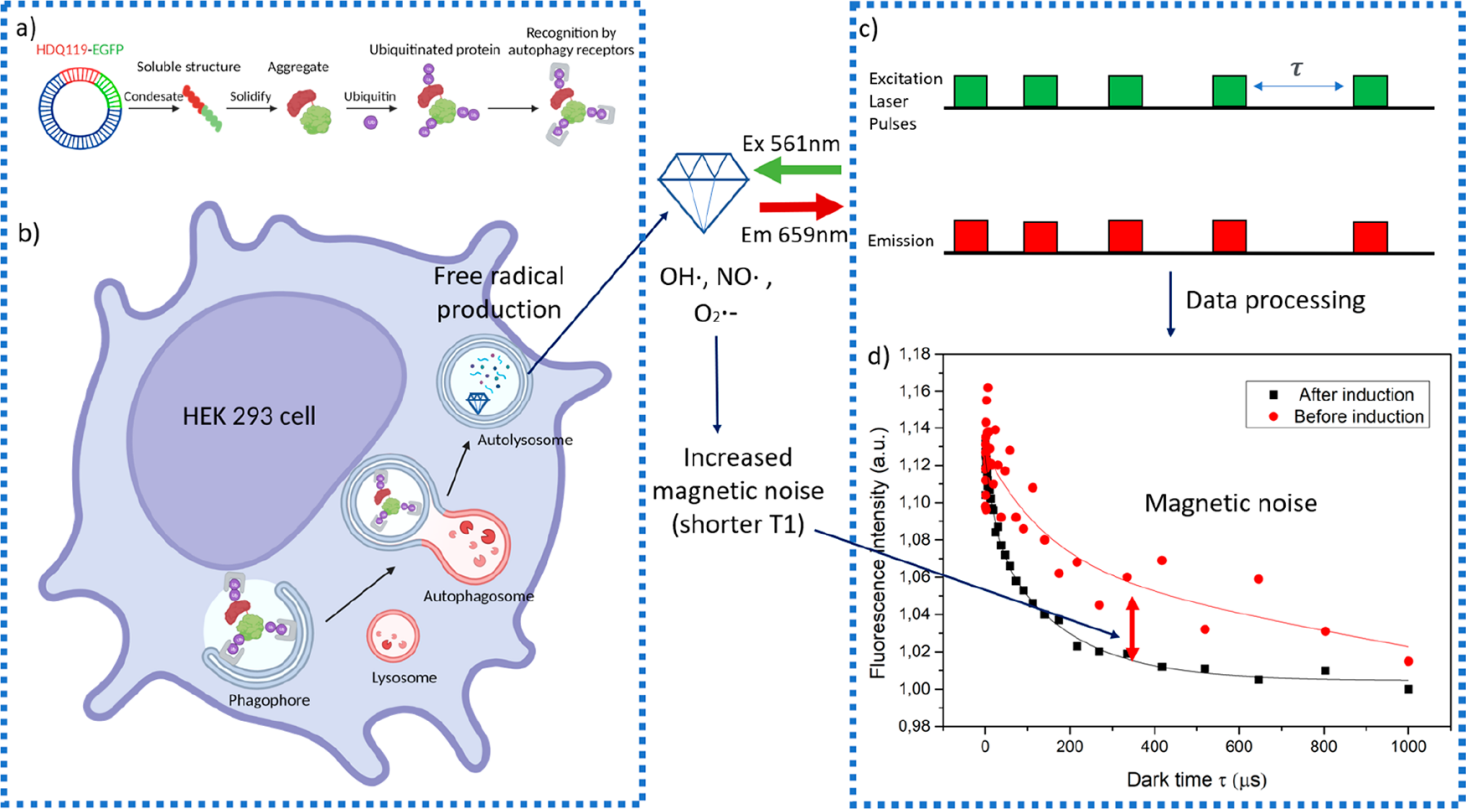
(a) EGFP-tagged mHtt proteins (PolyQ) are expressed after inducing cells by tetracycline (Tet). (b) The phagophores form around the polyQ aggregates to form autophagosomes that next fuse with lysosomes. After that, free radicals can be detected in autolysosomes. To measure T1 relaxation time, (c) a pulsing sequence of relaxometry measurements was used. Green laser pulses (561 nm) were applied, and during a variable dark time (τ) between 200 ns to 10 ms, the laser was turned off. The red blocks indicate photoluminescence when the signal from FNDs was read out within a fixed window to build the T1 relaxation curve. The NV^–^ centers in FNDs were pumped by the laser pulse to the bright m_s_ = 0 state of the ground state. The amount of NV centers that were still in the m_s_ = 0 state was examined after different dark times. A typical T1 measurement is shown in (d). The time needed to reach equilibrium is linked to the number of free radicals, with more free radicals leading to faster decay and a lower T1 value. The black line indicates faster relaxation due to magnetic noise in the environment when proteins and FNDs were colocalized with autolysosomes after induction. The pulsing sequence was repeated 10,000 times for each measurement to obtain a sufficient signal-to-noise ratio.

During T1 relaxometry measurements, we utilized the nitrogen-vacancy (NV) defect, which can be used for quantum sensing at room temperature. The NV centers in diamond can be used to read the magnetic noise of the surrounding region by optical means.^37^ The setup, which has been described earlier,^22^ is in principle a confocal microscope with an acousto-optical modulator (Gooch & Housego, model 3350–199) for detection. Using the pulse sequence shown in Figure 1a, we performed relaxometry and related the decay velocity to magnetic noise, which in this case stems from free radicals.

More specifically, the NV centers were excited with a train of 5 μs green laser pulses (561 nm) with a dark time (τ) between 200 ns to 10 ms (Figure 1a). The first 0.2 μs was used as the read window. The relaxometry curves showed the relaxation from the bright spin state to a darker equilibrium after different dark times (τ). The time needed to reach the equilibrium condition was linked to the presence of free radicals. The pulsing sequence was repeated 10,000 times for each measurement to gain a sufficient signal-to-noise ratio. The laser was attenuated to 50 μW at the location of the sample (measured at continuous illumination), which was chosen to minimize the damage to cells but be high enough to polarize the NV centers.

### Dihydroethidium (DHE) Assay Kit

HEK 293 cells (50,000 cells/well) were seeded in a clear flat-bottom 96-well plate. After incubating with 1 μg/mL tetracycline for 24, 36, or 48 h, cells were washed with PBS. The DHE solution (2 μg/mL) was prepared with DMEM medium and added to HEK 293 cells (200 μL/well) immediately after washing. Subsequently cells were incubated for 10 min at 37 °C and 5% CO_2_. DHE is a fluorescent probe used for the detection of ROS generation, specifically for intracellular superoxide and hydrogen peroxide. The fluorescence intensity was measured using a FLUOstar Omega Microplate Reader (BMG Labtech, De Meern, The Netherlands), with excitation and emission wavelengths at 514 and 580 nm. HEK WT cells and PQ cells were used as negative controls. The experimental protocol was followed according to the manufacturer’s manual.

### Statistical Analysis

Statistical analysis of all data was conducted using Graph pad prism version 8.0. Significance was tested by using the one-way or two-way ANOVA test according to different experiments. Significance was tested compared to the control group and defined as: ns *p* > 0.05, *p ≤ 0.05, **p ≤ 0.01, ***p ≤ 0.001, ****p ≤ 0.0001.

## Results and Discussions

First, we used confocal fluorescence microscopy to image the cells and determine the expression of PolyQ at different time points. Then, we quantified the proteins using various methods.

**Figure 2:**
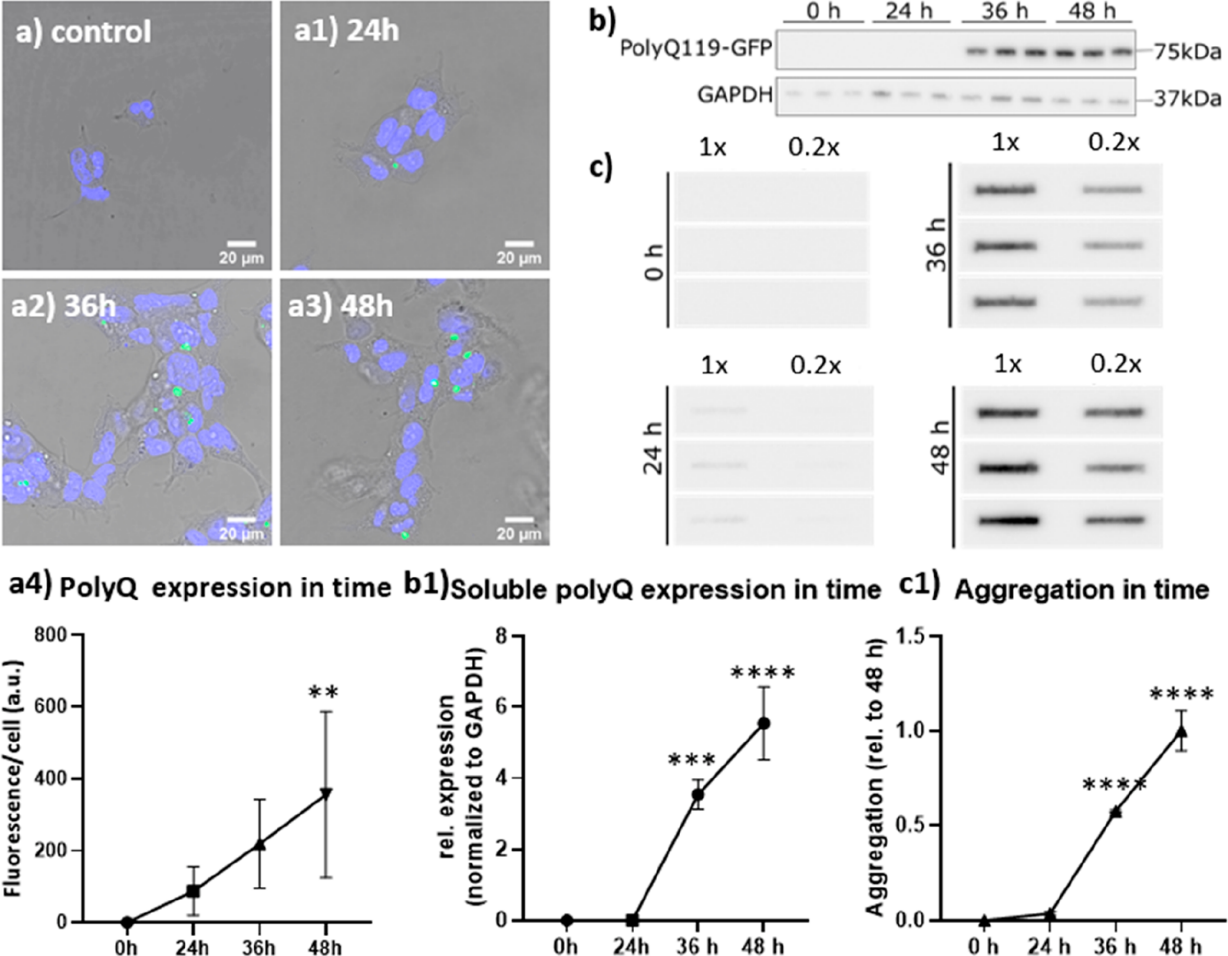
Validation of the EGFP Expression in HEK 293 cells. Representative images of (a) HEK 293 cell control (without inducer), or expressing PolyQ-EGFP after inducing by tetracycline for (a1) 24 h, (a2) 36 h, and (a3) 48 h, respectively. DAPI was used for nuclear staining. (a4) Quantitative analysis of EGFP by FIJI. (b) Expression of soluble polyQ119-GFP at different time points (same samples as in panel b). (b1) Representative WB (for panel b). (c) Measuring aggregation of polyQ119-GFP in time using a filter-trap assay (FTA). Each set of images represents an indicated time point. (c1) quantification of FTA from panel c. Error bars represent the standard deviations. The data were analyzed by using a one-way ANOVA and statistical difference is indicated by *p ≤ 0.05, **p ≤ 0.01, ***p ≤ 0.001, ****p ≤ 0.0001.

As previously reported,^7^ overexpressed PolyQ forms aggregates and accumulates in cells (Figure 2b–d). In this work, EGFP was conjugated with the PolyQ protein (Figure 1b). The strength of the fluorescence signal indicated the amount of both soluble proteins and aggregated PolyQ. The cells exhibited a continuous increase in fluorescence intensity with longer induction times. At 48 h, there was a significant difference compared to the control group (Figure 2a4). This observation was consistent with the measured amount of soluble proteins and aggregated PolyQ, both of which showed a significant increase at 36 and 48 h.

### Cellular Uptake of FNDs

**Figure 3:**
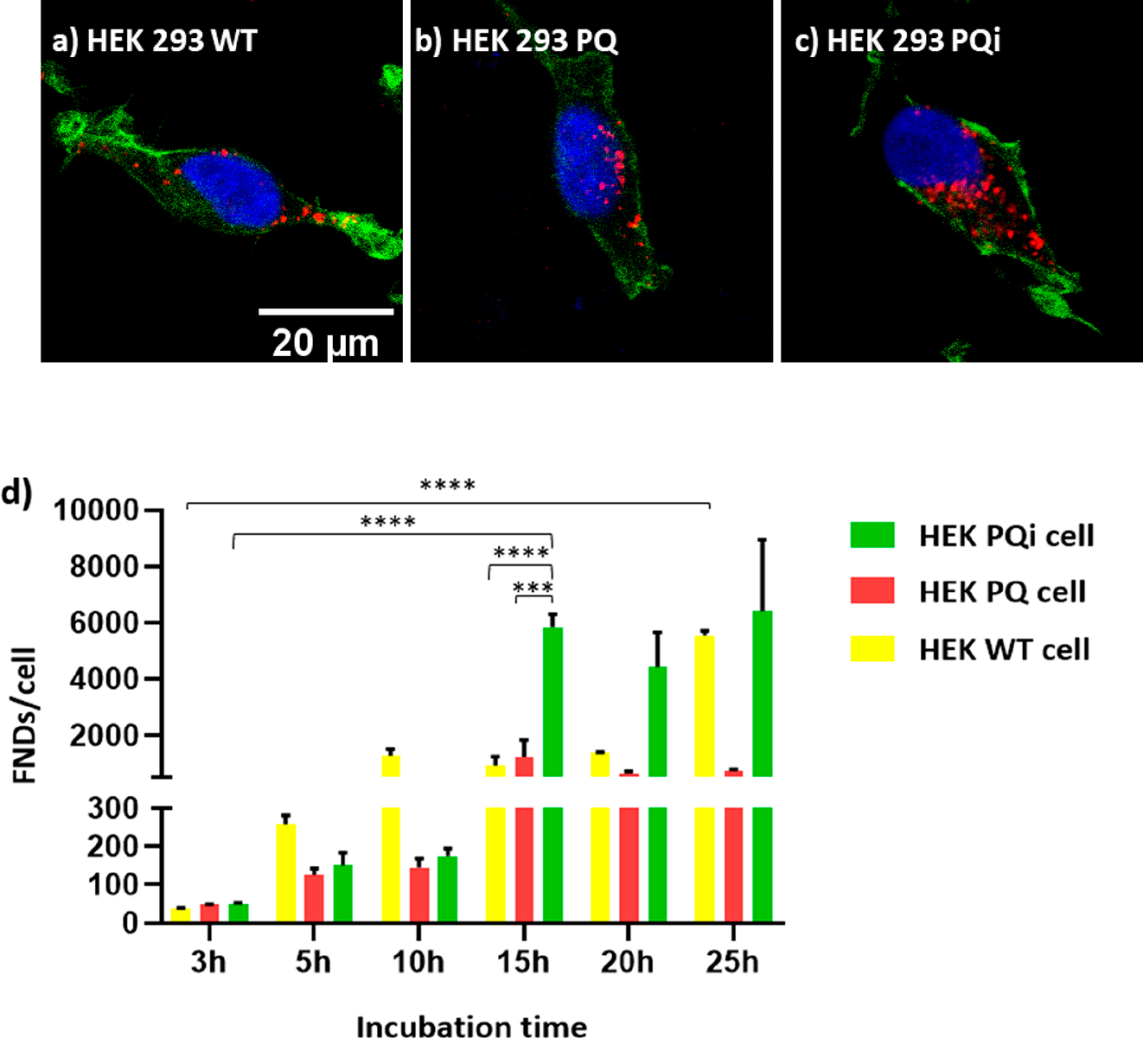
Diamond uptake by HEK 293 cells: HEK 293 WT cells (a), HEK 293 PQ cells (b), and HEK 293 PQi cells (c) were incubated with 10 μg/mL FNDs for 15 h. Color code: green, Phalloidin-FITC, staining actin filaments (also known as F-actin); blue: DAPI (staining DNA); red: FNDs. (d) Quantitative analysis of FND uptake per cell after incubation from 3 to 25 h. The experiment was repeated three times independently. Around 100 cells were counted in total for each cell type. Error bars represent the standard deviations. The data were analyzed by using a two-way ANOVA and statistical difference is indicated by *p ≤ 0.05, **p ≤ 0.01, ***p ≤ 0.001, and ****p ≤ 0.0001.

In order to perform relaxometry in cells, diamond particles should be internalized. As shown in Figure 3a to 3c, HEK 293 cells are able to ingest diamond particles and showed different uptake ability (Figure 3d). Confocal Z-stack images showed that, after 15 h of incubation, there were on average 918, 1244, and 5842 particles per cell in HEK WT, PQ, and PQi cell groups, respectively (Figure 3a–c). Some particles are slightly aggregated, but this was actually beneficial to spin measurements due to a slow-down in movement speed.

Initially, the number of FNDs inside HEK PQi cells showed a significant increase after 15 h of incubation, while for HEK WT cells, the number of FNDs peaked at the last time point (25 h). Although the uptake ability of HEK PQ cells was lower than that in other cells, the difference was not significant. As cells continued to incubate, they began to divide at a certain point, leading to diamonds inside cells being expelled or divided among cells during cell division. This could explain why the number of FNDs per cell stopped increasing after some time. It is crucial to maintain a relatively low FND density to avoid difficulties in tracking particles where a jump from one particle to another could be interpreted as a change in T1. Therefore, for further experiments, a 5 h incubation time was used due to the optimal particle density in all cell types at this time point.

### Colocalization of FNDs and Autolysosomes/PolyQ-GFP

To measure the free radical production inside cells, it is necessary to ensure that FNDs are present in the region of interest during relaxometry measurements. In this study, we investigated whether diamond particles colocalized with autolysosomes and PolyQ-GFP aggregates after a certain incubation time. Here, we used a 5 h incubation time for further experiments due to favorable particle density in all cell types at this time point.

**Figure 4:**
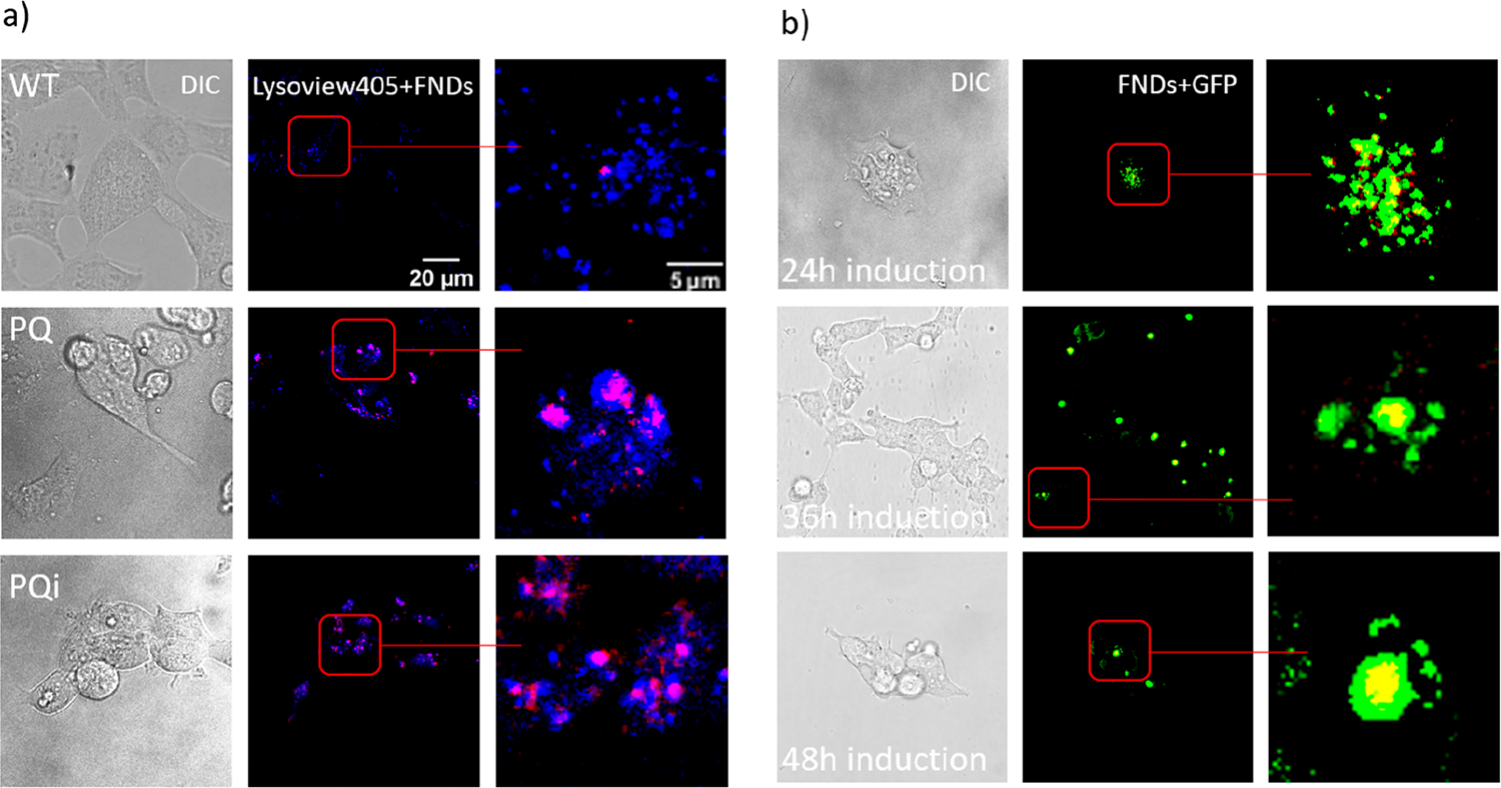
Subcellular location of FNDs and PolyQ-GFP was revealed by using a Zeiss 780 confocal microscope. (a) HEK 293 WT, HEK 293 PQ or HEK 293 PQi cells were incubated with FNDs for 5 h (HEK PQ cells were induced at 24 h to produce HEK PQi cells). (b) HEK PQ cells were induced by tetracycline for 24 h, 36 h, or 48 h to produce HEK PQi cells. Then, cells were stained and imaged. The location of autolysosomes inside HEK 293 cells was indicated by lysoview 405. Color code: green, PolyQ-GFP; red, FNDs; blue, autolysosome.

**Figure 5:**
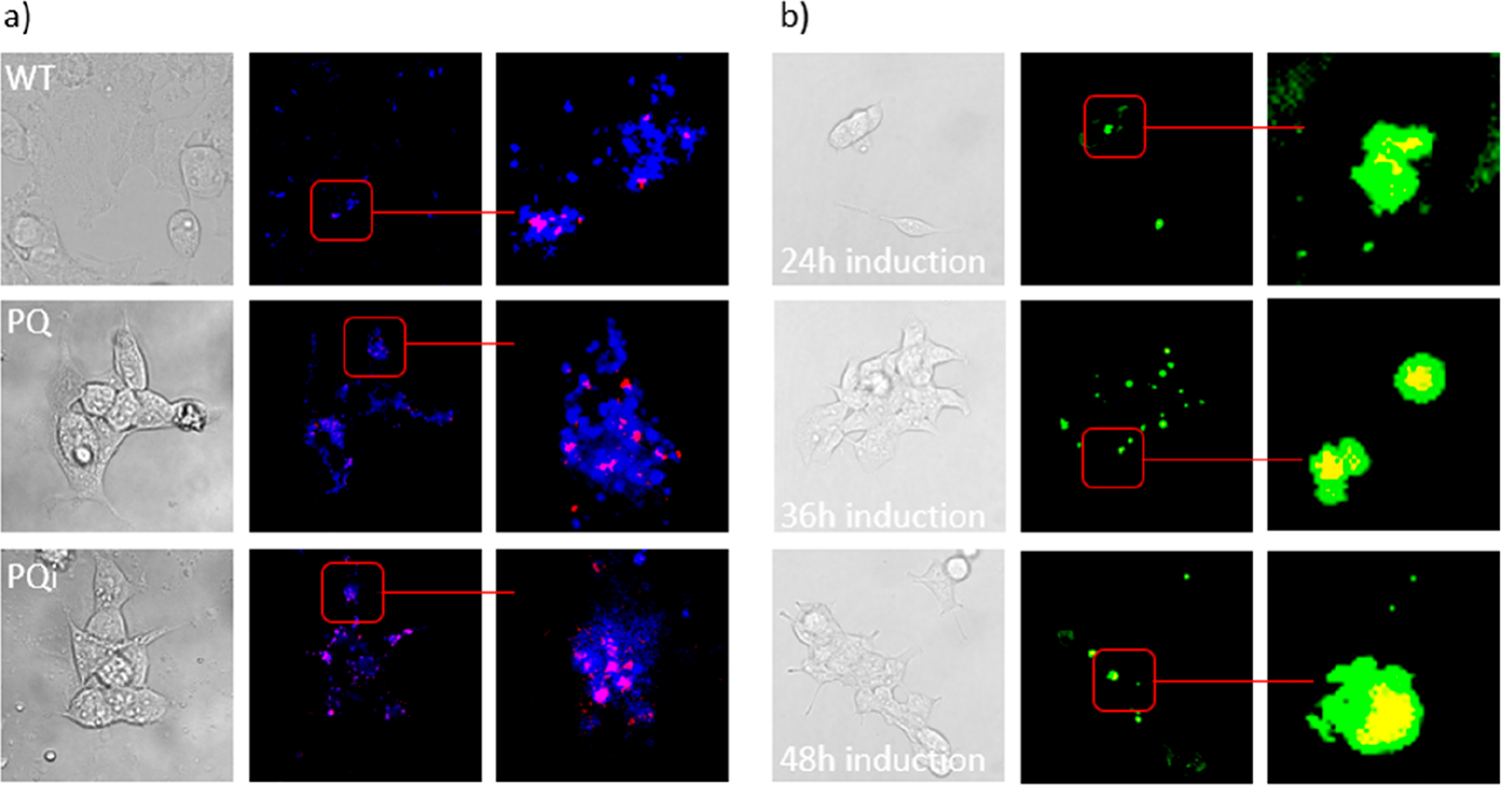
Subcellular locations of FNDs and PolyQ-GFP were revealed by using a Zeiss 780 confocal microscope. (a) HEK 293 WT cells, HEK 293 PQ cells, or HEK 293 PQi cells 5h+5h (HEK PQ cells were induced for 24 h to produce HEK PQi cells) were incubated with FNDs. 5h+5h meant FNDs were washed away after 5 h, and live cells were imaged after 5 more hours of incubation in FND-free medium. (b) HEK PQ cells were induced by tetracycline for 24 h, 36 h or 48 h to produce HEK PQi cells. Then, cells were imaged at 5h+5h. The location of autolysosomes inside HEK 293 cells was indicated by lysoview 405. Color code: green, PolyQ-GFP; red, FNDs; blue, autolysosome. Scale bar is the same as that in Figure 4.

Different HEK 293 cells were used for internalizing FNDs, and the intracellular location of these particles was evaluated by confocal Z-stack imaging (Figures 4 and 5). Figure 4a showed that FNDs highly colocalized with lysoview 405, indicating that FNDs were located at the autolysosomes after 5 h of incubation. In Figure 4b, FNDs were observed to be surrounded by PolyQ-GFP proteins. Large PolyQ-GFP protein aggregates were observed in the images after induction of cells for a long time. This probably led to high stress on autolysosomes.

We utilized Manders coefficients (MCs) to quantify the percentage of particles in autolysosomes (Tables 1 and 3) or PolyQ-GFP (Tables 2 and 4). MC is a widely used colocalization measurement that calculates the overlapping percentage of total signal from one channel to the other channel.^35,38^ As the number of FNDs was lower than the number of autolysosomes or PolyQ-GFP aggregates, we considered only M1, which is the fraction of FNDs in autolysosomes or PolyQ-GFP aggregates.

**Table 1:** Colocalization of Autolysosome and FND in Different Cells at the Start Point 5h+0h (from Panel a)^a^

Cell types	Mander’s Coefficient (MC)
HEK WT	0.97 ± 0.02
HEK PQ	0.97 ± 0.01
HEK PQi	0.97 ± 0.04

a Mander’s coefficients indicate the percentage of FNDs that are colocalized with autolysosomes. Error bars represent the standard deviations. The data were analyzed by using one-way ANOVA.

**Table 2:** Colocalization of FND and GFP in HEK PQi Cells at the Start Point 5h+0h (from Panel b)^a^

Induce time	Mander’s Coefficient (MC)
24 h	0.95 ± 0.03
36 h	0.92 ± 0.08
48 h	0.92 ± 0.08

a Mander’s coefficients indicate the percentage of FNDs which are colocalized with PolyQ-GFP. Error bars represent the standard deviations. The data were analyzed by using a one-way ANOVA.

The MC values for the three different HEK cell types are presented in Table 1. The MC values were high (ranging from 0.95 to 1.00) for all cell types, indicating that almost all endocytosed FNDs were at autolysosomes. Therefore, when performing T1 measurement after 5-hour incubation of FNDs, we could be confident that the location of FNDs was at autolysosomes.

In Table 2, we present the colocalization of FNDs and PolyQ-GFP. The MC values were close to 1.00 when cells were incubated for different times, indicating that almost all FNDs were colocalized with PolyQ proteins during T1 measurement.

The location of FNDs within the 5-hour time frame was also tracked by checking the end time point (5h+5h). We washed out the free FNDs in the cell culture medium after incubation for 5 h, then kept cell culturing for 5 more hours, and subsequently stained cells. As can be seen in Figure 5a and 5b, FNDs remained at the autolysosomes and polyQ-GFP after more 5 h of incubation.

The colocalization of FNDs with autolysosomes or polyQ-GFP at 5h+5h, were quantified in Table 3 and 4, respectively. Most of the FNDs still highly colocalized with autolysosomes or polyQ-GFP after 5 h.

**Table 3:** Colocalization of FNDs and Autolysosomes in Different Cells at the End Point 5h+5h (from Panel a)^a^

Cell types	Mander’s Coefficient (MC)
HEK WT	0.97 ± 0.03
HEK PQ	0.96 ± 0.03
HEK PQi	0.97 ± 0.02

a Mander’s coefficients indicate the percentage of FNDs which are colocalized with autolysosomes. Error bars represent the standard deviations. The data were analyzed by using a one-way ANOVA.

**Table 4:** Colocalization of FNDs and PolyQ-GFP in HEK PQi Cells at the End Point 5h+5h (from Panel b)^a^

Induce time	Mander’s Coefficient (MC)
24 h	0.97 ± 0.03
36 h	0.96 ± 0.03
48 h	0.97 ± 0.02

a Mander’s coefficients indicate the percentage of FNDs which are colocalized with PolyQ-GFP. Error bars represent the standard deviations. The data were analyzed by using a one-way ANOVA.

### Biocompatibility of FNDs for Cells

**Figure 6:**
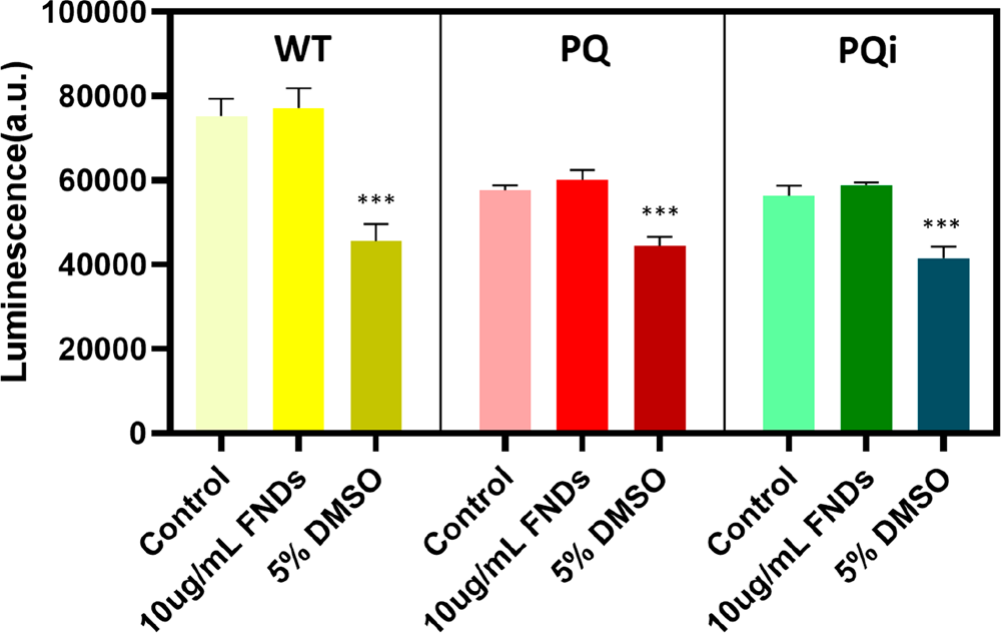
Cell viability test by a Cell Titer assay. 5% DMSO represents a positive control. The experiment was repeated for three different cells. Cells without FNDs were used as negative controls. Error bars represent the standard deviations. The data were analyzed by using a one-way ANOVA, and statistical difference is indicated by ***p ≤ 0.001.

To assess cell viability, we performed a Cell Titer assay (Figure 6) on different cell types incubated with 10 μg/mL FNDs or 5% DMSO for 24 h. As DMSO is known to induce cell death, it was used as a positive control. The results showed no significant difference in cell viability between the negative control and the cells exposed to FNDs, suggesting that FNDs exhibit good biocompatibility with HEK 293 cells. These findings are consistent with previous literature that has reported excellent biocompatibility of FNDs with various cell types.^39−41^

Potential influences on T1 by the inducer tetracycline have been ruled out (Figure S1) by testing the HEK WT cells which cannot be induced, and FNDs alone in solution in the presence of tetracycline. We did not see any significant differences under these conditions.

### Free Radical Measurements

**Figure 7:**
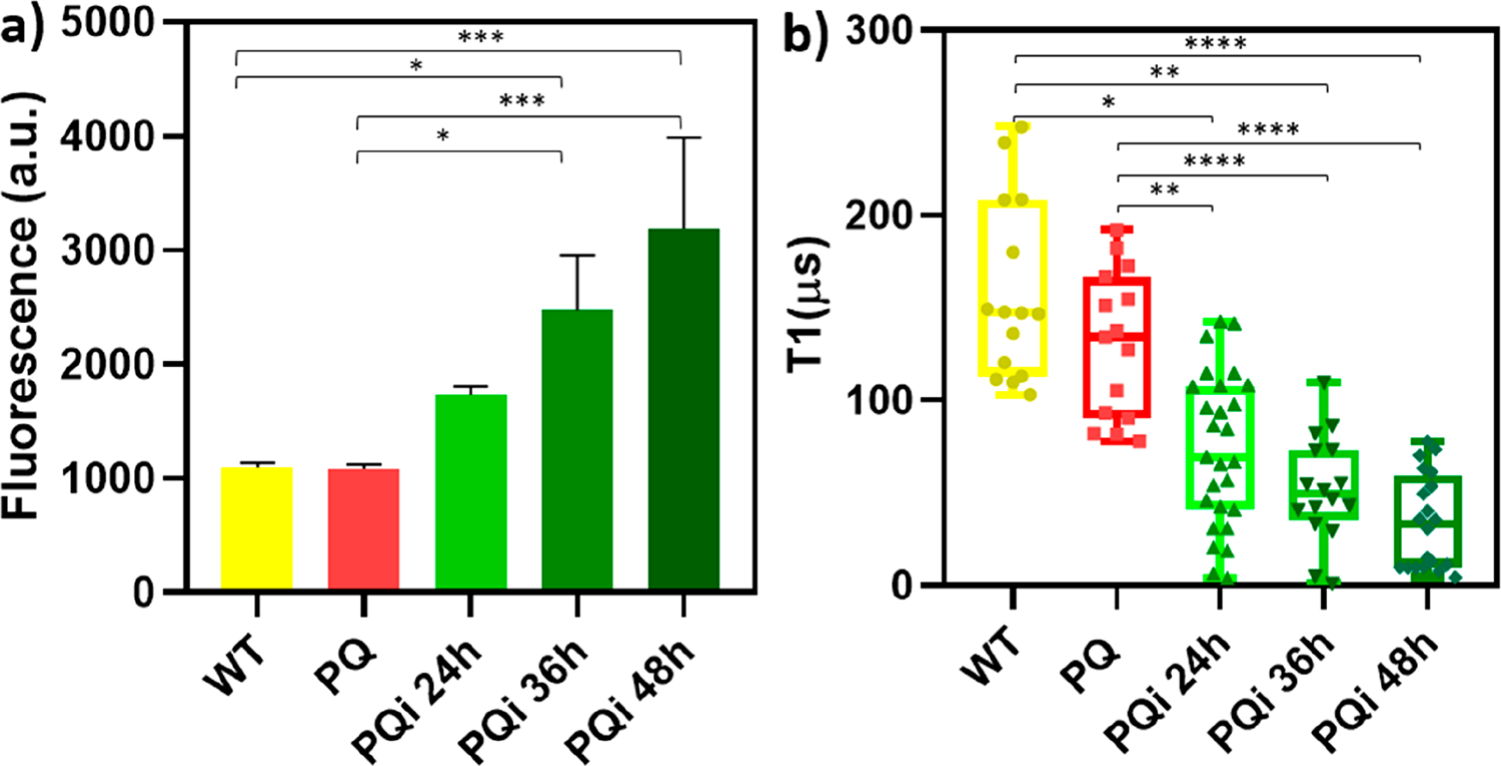
Free radical generation after inducing HEK PQ cells for 24, 36 or 48 h was determined by (a) the commercial Dihydroethidium (DHE) assay (specific for superoxide level detection) and (b) T1 measurements in single HEK 293 cells. Whiskers represent the lowest and highest data points. Data between the groups were analyzed by one-way ANOVA analysis: *p ≤ 0.05, **p ≤ 0.01, ***p ≤ 0.001, and ****p ≤ 0.0001.

DHE entered the cells and was oxidized by O_2_^•–^ to ethidium, which bonded to DNA, producing a red fluorescence. This probe is widely used to detect intracellular superoxide and hydrogen peroxide levels.^42,43^ When the induction time was extended, the amount of PolyQ-GFP increased (Figure 2). At the same time, the generation of free radicals was also observed (Figure 7a, b). When analyzing the difference in radical load by the DHE assay (Figure 7a), there was an increase after 24 h of inducing but no significant difference. A slight significant difference can be seen when culturing for 36 h. Incubating for longer time (48 h) led to a significantly higher free radical level. This finding agrees with reports in the literature where Winterbourn et al.^44^ found that superoxide is able to oxidize DHE to its radical. Thus, this finding was expected.

While the results of the DHE assay and the T1 measurements are similar, there are a few fundamental differences. When compared with our T1 results, which also exhibited significant differences of free radical levels after the different inducing times (Figure 7b), the DHE assay is less sensitive. The significant differences can be observed in every group. For each inducing time, the difference from the T1 results was bigger than that in the DHE results.

Besides the sensitivity, T1 measurements can be performed continuously, while the fluorescence from the DHE assay bleaches. While DHE measures the history of the sample, the T1 measurement reveals the current status. It is also worth noting that T1 measurements represent local information from the location of the autolysosomes (this does not exclude any radical production in other locations where we did not measure). Similarly, Nie et al. have shown that it is possible with this technique to measure locally on the surface of mitochondria.^23^ As a result, the T1 measurements confirm that free radical generation occurs in the autolysosomes, where polyQ is present. The DHE assay, on the other hand, provides measurements from a large ensemble of cells. Apart from that due to the different mechanisms of detection, T1 is prone to interferences from different factors than the conventional fluorescence assays. Fluorescence assays are prone to autoxidation of the probe or reactions with certain enzymes. Further interference with fluorescent molecules of the same wavelength influences the assay. These factors do not play a role in the T1 measurements. However, T1 measurements are influenced by drastic pH changes,^23^ paramagnetic ions (most critically iron), or spin labels in the surrounding.

The increase in the free radical level might be caused by stressed autolysosomes. When PolyQ proteins were accumulated, cellular excretion was affected, thus causing autolysosome storage disorder, leading to oxidative stress in autolysosomes. Autolysosomes play a central role in maintaining cellular homeostasis; once they are function-disrupted or damaged, this would result in progressive accumulation of partially degraded or nondegraded substrates in the autolysosomes and perturb the cellular homeostasis.^45^

## Conclusions

Relaxometry measurements are a powerful tool to assess free radical generation locally. Here, we have demonstrated this for measurements of the free radical load at autolysosomes where PolyQ accumulates in cells with the Huntington’s disease phenotype. As shown here, this method can be useful to gain a better understanding of the underlying mechanisms of an oxidative stress response. We were able to show that while we obtained a response similar to that with the conventional assay, the measurement was more sensitive, and it was possible to obtain the results in real time.

## Supporting Information Available

Controls where we performed T1 measurements while we (a) added the inducer to WT cells and (b) added the inducer to diamond particles in absence of cells (PDF)
Transparent Peer Review report available (PDF)
