# Supplementary material for: Diamond Quantum Sensing Revealing the Relation between Free Radicals and Huntington’s Disease
Source: ACS Cent Sci. 2023 Jun 21;9(7):1427–36. doi: 10.1021/acscentsci.3c00513 (PMC10375573; doi:10.1021/acscentsci.3c00513)
Supplement: Supplementary file 1 — oc3c00513_si_001.pdf [file oc3c00513_si_001.pdf]

Diamond quantum sensing revealing the relation between free radicals and Huntington's disease

S. Fan, L. Nie, Y. Zhang, E.I. Ustyantseva, W. Woudstra, H. Kampinga*, R. Schirhagl*

Groningen University, University Medical Center Groningen, Antonius Deusinglaan 1 9713AV Groningen, the Netherlands

Email: romana.schirhagl@gmail.com

Pages: 2

Figures: 1

Supplementary information

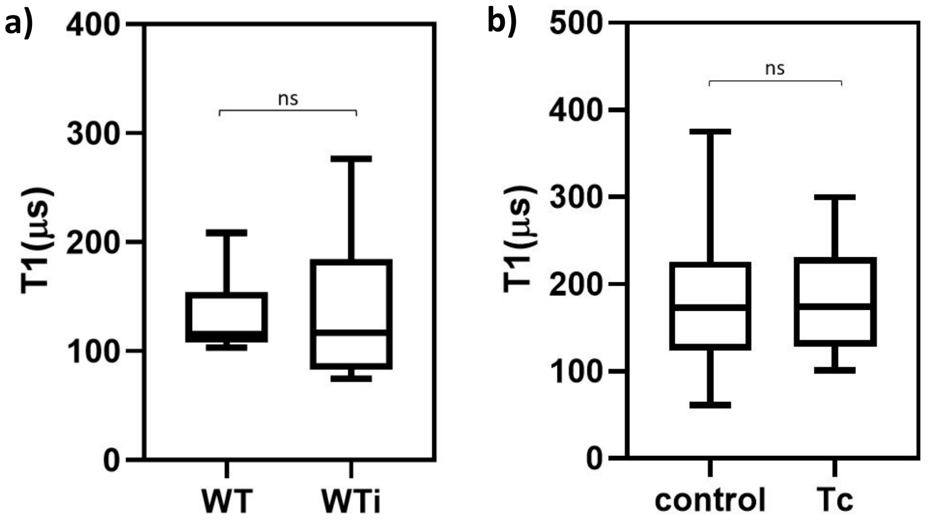

Figure S1 T1 measurement of a) HEK WT cells with inducer. b) FNDs solution with inducer. Whiskers represent from the highest and lowest data point, data between each group were analyzed by one-way ANOVA analysis: ns, no significant difference.
