# Supplementary material for: Diamond Quantum Sensing Revealing the Relation between Free Radicals and Huntington’s Disease
Source: ACS Cent Sci. 2023 Jun 21;9(7):1427–36. doi: 10.1021/acscentsci.3c00513 (PMC10375573; doi:10.1021/acscentsci.3c00513)
Supplement: Supplementary file 2 — oc3c00513_si_002.pdf [file oc3c00513_si_002.pdf]

oc-2023-005135.R1

Name: Peer Review Information for "Diamond quantum sensing revealing the relation between free radicals and Huntington's disease"

First Round of Reviewer Comments

Reviewer: 1

Comments to the Author

Diamond quantum sensing revealing the relation between free radicals and Huntington's disease

The article (oc-2023-005135 is about a new kind of quantum sensing technique called relaxometry which is used here for the first time to investigate the link between Huntington protein aggregate formation and free radical generation. In this context the new method has the advantage that it allows location specific measurements in the vesicles here Poly-Q also accumulate. The article is well written and clear and deserves publication. Below is a list of my minor criticism.

1) "The formation of aggregates by PolyQ leads to the disease and increases the level of free radicals" should be "The formation of aggregates of PolyQ leads to the disease and increases the level of free radicals."
2) "The CAG repeat in HTT leads to an expansion of the polyglutamine (PolyQ) tract at the N-terminus of huntingtin (HTT), which can become an amyloid core and induces toxic protein aggregation." HTT was already defined above and doesn't need to be defined here again.
3) The caption of Fig 1 is formatted slightly differently.
4) "The phagophores form around the polyQ aggregates to make autophagosomes that next fuse with lysosomes after which free radicals can be detected in autolysosomes." I would recommend splitting this sentence as it is difficult to read.
5) "The black line indicated faster relaxation due to magnetic noise in the environment when proteins and FNDs were colocalized with autolysosomes after induction." Should be "The black line indicates faster relaxation due to magnetic noise in the environment when proteins and FNDs were colocalized with autolysosomes after induction."
6) "These particles were generated by high-temperature high pressure (HPHT) synthesis followed by irradiation and high-temperature annealing." I am aware that it is a commercial product but can you give the conditions for irradiation and annealing?
7) "Larger FNDs were preferred due to their brightness and ease of tracking, resulting in a good signal-to-noise ratio. In addition, each measurement is an average of all the NV^- centers." Too large particles are also not ideal for these kinds of experiments. This should also be mentioned.

8) “Stable tetracycline (tet)-inducible HDQ119-EGFP-expressing cell types (HEK PQ) were produced as shown in reference” should be “Stable tetracycline (tet)-inducible HDQ119-EGFP-expressing cells (HEK PQ) were produced as shown in reference.”

9) “The cells were co-transfected with pcDNA5/FRT/TO HDQ119-EGFP and the flippase (Flp) recombinase expressing plasmid pOG44 and selected with 100 mg/ml hygromycin.” Here it should be mL.

10) “For HEK 293 wild type cells (HEK WT), once per week fresh Blasticidine (Invitrogen) and week fresh Zeocin (Invitrogen) were added to the culture medium to a final concentration of 5 μg/mL and 100 μg/mL, respectively.” What is meant here by “and week fresh Zeocin (Invitrogen) were” I think there is some grammatical error.

11) “For culturing HEK cells containing PolyQ plasmid (HEK PQ), fresh Blasticidine (Invitrogen, 5 μg/mL) and Hygromycin B (Invitrogen, 100 μg/mL) were added to the culture medium once per week.” Should be “For culturing HEK cells containing PolyQ plasmids (HEK PQ), fresh Blasticidine (Invitrogen, 5 μg/mL) and Hygromycin B (Invitrogen, 100 μg/mL) were added to the culture medium once per week.”

12) “As HEK 293 cells do not adhere very well to the glass bottom Petri-dish, 0.2% gelatin (Merck) was applied to coat the dishes prior to introducing HEK 293 cells to glass bottom Petri-dish.” Here the glass bottom Petri dishes appear twice so it is a bit redundant. Consider rephrasing.

13) “The PolyQ protein was tagged with GFP; the green fluorescence of GFP can indicate the success of PolyQ expression.” Consider revising the grammar here.

14) “A control group was used to substract the background for other groups.” There is a typo in subtract.

15) “The resulting graph represent an average of three separate experiments.” Should be “The resulting graph represents an average of three separate experiments.”

16) “Z- stack confocal images containing the whole volume were acquired and deconvolved.” The whole volume of what? The cells?

17) “This number had been determined earlier from a control group to separate FND signal from background.: should be “This number has been determined earlier from a control group to separate the FND signal from the background.”

18) “Then inducer was washed away and FNDs were added to the cell culture medium.” Should be “Then the inducer was washed away and FNDs were added to the cell culture medium.”

19) “Measuring aggregation of polyQ119-GFP in time using filter-trap assay (FTA).”

20) Should be “Measuring aggregation of polyQ119-GFP in time using a filter-trap assay (FTA).”

21) “This observation was consistent with the quantification results of soluble proteins and aggregated PolyQ, both of which showed a significant increase at 36 and 48 hours.” This sentence should be revised.

22) “Although the uptake ability of HEK PQ cells was lower than other cells, it was not significant.” Should be “Although the uptake ability of HEK PQ cells was lower than in other cells, the difference was not significant.”

23) “As cells continued to incubate, they began to divide at a certain point, leading to diamonds inside cells being expelled or going outside along with cell division.” This sentence should be revised.

24) “Subcellular location of FNDs and PolyQ-GFP were revealed by using a Zeiss 780 confocal microscope” should be “The subcellular location of FNDs and PolyQ-GFP were revealed by using a Zeiss 780 confocal microscope.”

25) “Colocalization of autolysosome and FND in different cells at start point 5h+0h” should be “Colocalization of autolysosome and FND in different cells at 5h+0h”

26) “FNDs were incubated with HEK 293 WT cells, HEK 293 PQ cells or HEK 293 PQi cells 5h+5h (HEK PQ cells were induced” it is not Particles that are incubated with cells but should be cells that are incubated with particles.

27) “Colocalization of FNDs and autolysosome FND in different cells at the endpoint 5h+5h (From panel a).” I think there is a grammatical error here.

28) “Whiskers represent the lowerst and highest data points.” There is a typo here.

29) “As a result, the T1 measurements confirm that free radical generation occurs in the autolysosomes where polyQ present” should be “As a result, the T1 measurements confirm that free radical generation occurs in the autolysosomes where polyQ is present.”

Author's Response to Peer Review Comments:

Dear editors and reviewers,

First of all, we would lie to than you for your valuable time and for helping us improve our manuscript. We have made the suggested changes and we hope that the new submission meets your expectations. Below you can find the point by point response to the reviewer and editor comments. Further, the changes are marked in yellow in the revised manuscript.

Best regards

Romana Schirhagl

Diamond quantum sensing revealing the relation between free radicals and Huntington's disease The article (oc-2023-005135 is about a new kind of quantum sensing technique called relaxometry which is used here for the first time to investigate the link between Huntington protein aggregate formation and free radical generation. In this context the new method has the advantage that it allows location specific measurements in the vesicles here Poly-Q also accumulate. The article is well written and clear and deserves publication. Below is a list of my minor criticism.

1) "The formation of aggregates by PolyQ leads to the disease and increases the level of free radicals" should be "The formation of aggregates of PolyQ leads to the disease and increases the level of free radicals."

We have followed the advice of the reviewer and corrected the sentence.

2) "The CAG repeat in HTT leads to an expansion of the polyglutamine (PolyQ) tract at the N-terminus of huntingtin (HTT), which can become an amyloid core and induces toxic protein aggregation." HTT was already defined above and doesn't need to be defined here again.

We reviewer is right. We have removed the abbreviation here.

3) The caption of Fig 1 is formatted slightly differently.

We have changed the formatting of the caption.

4) "The phagophores form around the polyQ aggregates to make autophagosomes that next fuse with lysosomes after which free radicals can be detected in autolysosomes." I would recommend splitting this sentence as it is difficult to read.

We have split this sentence to improve readability.

5) "The black line indicated faster relaxation due to magnetic noise in the environment when proteins and FNDs were colocalized with autolysosomes after induction." Should be "The black line indicates faster relaxation due to magnetic noise in the environment when proteins and FNDs were colocalized with autolysosomes after induction."

We have followed the reviewer's suggestion and made the changes.

6) "These particles were generated by high-temperature high pressure (HPHT) synthesis followed by irradiation and high-temperature annealing." I am aware that it is a commercial product but can you give the conditions for irradiation and annealing?

We have added the conditions that are given by the manufacturer and referred to the relevant literature.

7) "Larger FNDs were preferred due to their brightness and ease of tracking, resulting in a good signal-to-noise ratio. In addition, each measurement is an average of all the NVcenters." Too large particles are also not ideal for these kinds of experiments. This should also be mentioned.

This is correct. We have added the missing statement.

8) "Stable tetracycline (tet)-inducible HDQ119-EGFP-expressing cell types (HEK PQ) were produced as shown in reference" should be "Stable tetracycline (tet)-inducible HDQ119-EGFP-expressing cells (HEK PQ) were produced as shown in reference."

We have changed the sentence as requested.

9) "The cells were co-transfected with pcDNA5/FRT/TO HDQ119-EGFP and the flippase (Flp) recombinase expressing plasmid pOG44 and selected with 100 mg/ml hygromycin." Here it should be mL.

We have corrected the error.

10) "For HEK 293 wild type cells (HEK WT), once per week fresh Blasticidine (Invitrogen) and week fresh Zeocin (Invitrogen) were added to the culture medium to a final concentration of 5 µg/mL and 100 µg/mL, respectively." What is meant here by "and week fresh Zeocin (Invitrogen) were" I think there is some grammatical error.

We have revised the sentence to correct the grammar.

11) "For culturing HEK cells containing PolyQ plasmid (HEK PQ), fresh Blasticidine (Invitrogen, 5 µg/mL) and Hygromycin B (Invitrogen, 100 µg/mL) were added to the culture medium once per week." Should be "For culturing HEK cells containing PolyQ plasmids (HEK PQ), fresh Blasticidine (Invitrogen, 5 µg/mL) and Hygromycin B (Invitrogen, 100 µg/mL) were added to the culture medium once per week."

We have made the correction that was suggested by the reviewer.

12) "As HEK 293 cells do not adhere very well to the glass bottom Petri-dish, 0.2% gelatin (Merck) was applied to coat the dishes prior to introducing HEK 293 cells to glass bottom

Petri-dish." Here the glass bottom Petri dishes appear twice so it is a bit redundant. Consider rephrasing.

We have rephrased this text passage as suggested.

13) "The PolyQ protein was tagged with GFP; the green fluorescence of GFP can indicate the success of PolyQ expression." Consider revising the grammar here.

Considering this suggestion, we have revised the grammar here.

14) "A control group was used to substract the background for other groups." There is a typo in subtract.

We have corrected the typo.

15) "The resulting graph represent an average of three separate experiments." Should be "The resulting graph represents an average of three separate experiments."

We have made the suggested change.

16) "Z- stack confocal images containing the whole volume were acquired and deconvolved." The whole volume of what? The cells?

Yes. Here we meant the whole volume of the cells. We have revised this sentence to make this more clear.

17) "This number had been determined earlier from a control group to separate FND signal from background.: should be "This number has been determined earlier from a control group to separate the FND signal from the background."

We have made the suggested edit to improve the sentence.

18) "Then inducer was washed away and FNDs were added to the cell culture medium." Should be "Then the inducer was washed away and FNDs were added to the cell culture medium."

We have made the suggested correction.

19) "Measuring aggregation of polyQ119-GFP in time using filter-trap assay (FTA)." 20) Should be "Measuring aggregation of polyQ119-GFP in time using a filter-trap assay (FTA)."

We have followed this suggestion and edited the text.

21) "This observation was consistent with the quantification results of soluble proteins and aggregated PolyQ, both of which showed a significant increase at 36 and 48 hours." This sentence should be revised.

Following this suggestion, we have revised this sentence.

22) “Although the uptake ability of HEK PQ cells was lower than other cells, it was not significant.” Should be “Although the uptake ability of HEK PQ cells was lower than in other cells, the difference was not significant.”

We have changed the sentence as requested.

23) “As cells continued to incubate, they began to divide at a certain point, leading to diamonds inside cells being expelled or going outside along with cell division.” This sentence should be revised.

To improve clarity, we have revised this sentence.

24) “Subcellular location of FNDs and PolyQ-GFP were revealed by using a Zeiss 780 confocal microscope” should be “The subcellular location of FNDs and PolyQ-GFP were revealed by using a Zeiss 780 confocal microscope.”

We have followed the suggested and rephrased this sentence.

25) “Colocalization of autolysosome and FND in different cells at start point 5h+0h” should be “Colocalization of autolysosome and FND in different cells at 5h+0h”

Following this suggestion by the reviewer, we have corrected the text.

26) “FNDs were incubated with HEK 293 WT cells, HEK 293 PQ cells or HEK 293 PQi cells 5h+5h (HEK PQ cells were induced” it is not Particles that are incubated with cells but should be cells that are incubated with particles.

This is correct. We have corrected this mistake.

27) “Colocalization of FNDs and autolysosome FND in different cells at the endpoint 5h+5h (From panel a).” I think there is a grammatical error here.

We have corrected the grammatical error.

28) “Whiskers represent the lowerst and highest data points.” There is a typo here.

We have corrected the typo.

29) “As a result, the T1 measurements confirm that free radical generation occurs in the autolysosomes where polyQ present” should be “As a result, the T1 measurements confirm that free radical generation occurs in the autolysosomes where polyQ is present.”

We have made the requested change.

Editorial comments:

AU EMAIL: Please include the email address of the corresponding author on the first page of the manuscript, and the Supporting Information if submitted, with an asterisk next to their name in the author list. Please be sure to label “email.”

We have added the corresponding author emails.

REFERENCES: Please remove double

We have removed any duplicate references.

SI PARAGRAPH: If the manuscript is accompanied by any supporting information for publication, a brief description of the supplementary material is required in the manuscript. The appropriate format is: Supporting Information. Brief statement in non-sentence format listing the contents of the material supplied as Supporting Information.

We have added the SI paragraph.

GENERAL REF FORMATTING: Periodical references should contain authors’ surnames followed by initials, article title, journal abbreviation, year, volume number, and page range. Refs with more than 10 authors should list the first 10 and then be followed by “et al.”

We have adapted the reference formatting.

Web sources must include access date.

Following this suggestion we have added the access dates.

TOC MISSING: Provide a TOC image per journal guidelines (3.25 in. × 1.75 in. (8.25 cm × 4.45 cm) ; on the last page of the Manuscript) with the heading “TOC Graphic” above the graphic. Make sure to designate the file as “Graphic for Manuscript.”

We have added a TOC graphic.

SYNOPSIS MISSING: The synopsis should be no more than 200 characters (including spaces) and should reasonably correlate with the TOC graphic. The synopsis is intended to explain the importance of the article to a broader readership across the sciences. Please place your synopsis in the manuscript file after the TOC graphic.

We have added a synopsis to complement the TOC graphic.

SI HEADER: The supporting information should be formatted with a cover sheet listing authors, author affiliations, corresponding author email, manuscript title, and the number of pages, figures, and tables. The Author affiliations must match the MS.

As requested, we have added a the title page.

SI PG#S: The supporting information pages must be numbered consecutively, starting with page S1.

As requested we have added page numbers in the SI.
